# Genome-Wide Association Studies of Salt-Alkali Tolerance at Seedling and Mature Stages in *Brassica napus*

**DOI:** 10.3389/fpls.2022.857149

**Published:** 2022-04-27

**Authors:** Guofang Zhang, Yan Peng, Jinzhi Zhou, Zengdong Tan, Cheng Jin, Shuai Fang, Shengzhu Zhong, Cunwang Jin, Ruizhen Wang, Xiaoliang Wen, Binrui Li, Shaoping Lu, Guangsheng Zhou, Tingdong Fu, Liang Guo, Xuan Yao

**Affiliations:** ^1^National Key Laboratory of Crop Genetic Improvement, Huazhong Agricultural University, Wuhan, China; ^2^Hubei Hongshan Laboratory, Wuhan, China; ^3^Agriculture and Animal Husbandry Technology Promotion Center, Inner Mongolia, China; ^4^Green Industry Development Center, Inner Mongolia, China

**Keywords:** GWAS, salt-alkali stress, seedling stage, mature stage, *Brassica napus*

## Abstract

Most plants are sensitive to salt-alkali stress, and the degree of tolerance to salt-alkali stress varies from different species and varieties. In order to explore the salt-alkali stress adaptability of *Brassica napus*, we collected the phenotypic data of 505 *B. napus* accessions at seedling and mature stages under control, low and high salt-alkali soil stress conditions in Inner Mongolia of China. Six resistant and 5 sensitive materials, respectively, have been identified both in Inner Mongolia and Xinjiang Uygur Autonomous Region of China. Genome-wide association studies (GWAS) for 15 absolute values and 10 tolerance coefficients (TCs) of growth and agronomic traits were applied to investigate the genetic basis of salt-alkali tolerance of *B. napus*. We finally mapped 9 significant QTLs related to salt-alkali stress response and predicted 20 candidate genes related to salt-alkali stress tolerance. Some important candidate genes, including *BnABA4, BnBBX14, BnVTI12, BnPYL8*, and *BnCRR1*, were identified by combining sequence variation annotation and expression differences. The identified valuable loci and germplasms could be useful for breeding salt-alkali-tolerant *B.napus* varieties. This study laid a foundation for understanding molecular mechanism of salt-alkali stress adaptation and provides rich genetic resources for the large-scale production of *B. napus* on salt-alkali land in the future.

## Introduction

Salt-alkali stress has been considered as a major environmental threat to the entire terrestrial ecosystem, which is one of the main reasons to inhibit the growth, development, and even the yield of crops in the world (Lin et al., 2014, 2018a,b; Guo et al., 2015). Although there are many ways to improve crop tolerance to salt-alkali stress (Sanchez-Bel et al., 2012), there is still a challenge to maintain the food supply as the population of the world continues to increase (Hickey et al., 2019; Tyerman et al., 2019). It is an important step to increase crop agricultural productivity through in-depth understanding of the genetic and molecular mechanisms for improving crop salt-alkali tolerance.

Soil salinization is a universal environmental problem worldwide. According to the incomplete statistics of the Food and Agriculture Organization of the United Nations, the area of salt-affected land in the world is as high as 9.54 × 10^8^ hm^2^ (Munns and Tester, 2008; Hussain et al., 2019; Singh et al., 2020). The distribution area of salt-alkali soil in China is very wide (Wang et al., 2011). According to the estimation from the Ministry of Agriculture and Rural Affairs of China, it is about 20-million-hectare salt-alkali lands (Song and Liu, 2017; Xu et al., 2020), accounting for one fourth of the total area of the cultivated land in China. Saline sodic soil is the most widely distributed soil type of semi-arid areas. Northwestern, northeastern, and central China, especially Xinjiang and Inner Mongolia Autonomous Region of China, are typical arid areas, where soil salinization is very serious and a large area of saline-alkali land has been formed (Lin, 2005; Jin et al., 2008; Lin et al., 2016). It not only becomes a major obstacle to the development of local agriculture but also an important factor affecting the ecological stability. Such a large area of salt-alkali soil has a great impact on agricultural production of China, especially for large-scale mechanized planting and industrial production (Pang et al., 2016).

Salt-alkali stress actually includes two abiotic stresses, including salt stress and high pH value (Zhou and Yang, 2003; Fang et al., 2021), which causes osmotic stress, ion toxicity, high pH value, and other hazards to plants (Huan, 2015). When the salt content reaches 0.2% or pH reaches 8., salt-alkali stress will result in a serious harm to plants (Chen et al., 2018). When the salt ions and pH value in the soil are too high, it will seriously affect the water potential balances in the plant roots, thereby affecting the absorption and transportation of water, and indirectly affecting the transportation and absorption of plant nutrients, which severely restricts the normal growth and development of plants and affects crop yield (Zhu, 2002; Julkowska and Testerink, 2015). When plants are subjected to short-term salt-alkali stress, a higher osmotic pressure will cause cell membrane damages, thereby leading to water loss and showing leaf wilting (Khan and Weber, 2006; Chikelu et al., 2007; Munns and Tester, 2008). Long-term salt-alkali stress causes the imbalance of ion absorption and ion transport, resulting in nutritional imbalance and metabolic disorders. This will inhibit the growth inhibition of new leaves, early senescence, and shedding of old leaves (Zhu, 2001). The plants themselves can resist salt-alkali stress to ensure their normal growth and development to some extent. However, when the salt-alkali stress level is greater than the resistance of the plant, the normal growth and the development of the plant are restricted (Munns and Tester, 2008; Julkowska and Testerink, 2015). The regulation mechanism of plants to salt-alkali stress mainly includes osmotic regulation, an active oxygen scavenging mechanism, ion regional regulation, and selective absorption of ions (Dwivedi, 2004; Zhu, 2016; Sanders, 2020).

*Brassica napus* has an important economic value of oil use, feed use, vegetable use, and tourism (Lu et al., 2019; Song et al., 2020). Previous studies have revealed that overexpression of *AtNHX1* and *BnaABF2* could significantly improve salt tolerance in transgenic *B. napus* and *Arabidopsis*, respectively (Zhang et al., 2001; Zhao et al., 2016). Using RNA-seq, 582 transcription factors and 438 transporter genes have been identified by analyzing differential expression genes (DEGs) under salt stress (Yong et al., 2014). Moreover, using the linkage analysis, 9 QTLs were mapped on Chromosomes A02, A04, and C03 with recombinant inbred lines (RIL) population from GH06 and P174 using the linkage analysis (Hou et al., 2017). Genome-wide association study (GWAS) is an effective way to identify QTLs related to complex traits for crops, which can improve efficiency and cost savings to parse the genetic basis based on abundant genetic variation (Pearson and Manolio, 2008; Huang et al., 2012; De et al., 2014; Patishtan et al., 2017). In recent years, some QTLs and candidate genes related to salt stress response have gradually been identified through GWAS. About 225 significant SNPs (He et al., 2017), 44 QTLs including 56 possible candidate genes (Wan et al., 2018), and 65 candidate genes (Zhang et al., 2017) related to salt stress response of *B. napus* were identified by GWAS using the 60K Brassica Illumina^®^ Infinium SNP array at the germination stage, respectively. In addition, 25 QTLs, including 38 promising candidate genes associated with salt stress tolerance at the seedling stage, were also identified by GWAS using the 60k *Brassica* Infinium^®^ SNP array in 368 *Brassica* accessions (Wan et al., 2017). Based on the next-generation sequencing technology and GWAS approach, a total of 62 QTLs were identified for salt tolerance index, growth indicators, and Na^+^/K^+^ ratio traits at the seedling stage in 85 rapeseed inbred lines (Yong et al., 2015). About 142 significant SNP markers and 117 possible candidate genes associated with salt tolerance of *B. napus* were also identified for SSI and STI values using 201,817 high-quality SNPs (Wassan et al., 2021). In addition, our previous study has revealed that a total of 177 and 228 candidate genes related to salt stress tolerance were identified at germination and seedling stages, respectively, and some important candidate genes, such as *BnCKX5* and *BnERF3*, which were found to play a positive role in salt and mannitol stress response at the germination stage (Zhang et al., 2022). Although a great number of QTLs and candidate genes related to salt stress have been identified, the molecular mechanism for salt-alkali tolerance of *B. napus* remains unclear (Chen et al., 2021). Moreover, the experiments conducted in the greenhouse cannot completely reflect the true response of plants to salt-alkali stress. Therefore, it is of great significance to carry out field trials on salt-alkali lands.

In this study, 11 extreme materials have been screened from 505 *B. napus* accessions grown on the salt-alkali lands of Inner Mongolia and Xinjiang Uygur Autonomous Region of China. We also performed GWAS for 15 absolute values and 10 tolerance coefficients (TCs) of some growth and agronomic traits in 505 *B. napus* accessions under salt-alkali stress to identify main-effect QTLs and candidate genes associated with salt-alkali stress response. This study will help us to reveal the genetic mechanism of salt-alkali stress adaptation and provide a valuable reference to genetic improvement in salt-alkali tolerance to *B. napus*.

## Materials and Methods

### Materials

These materials consisting of 505 natural accessions of *B. napus* (Supplementary Table 1) (Tang et al., 2021) were used as research objects under control, low salt-alkali, and high salt-alkali stress conditions at seedling and mature stages.

### Determination of Salt Content and pH Value of Salt-Alkali Land

Eight spots covering the entire salt-alkali land in Inner Mongolia of China were randomly selected, and 3 soil samples (10–20-cm depth) were taken in within the area (50–100 m^2^) of each spot. After the dried samples were filtered with a 1-mm sieve, 100 g of the filtered samples were transferred to a 1-L plastic bottle, and 500-ml carbon dioxide distilled water was added into the bottle (Water: Soil = 5:1). Suction and filtration were immediately performed after shaking for 8 min. We put 50–100 ml of the test solution into an evaporating dish with known weight, and then evaporated the evaporating dish to dryness. We wiped the outside of the evaporating dish with a filter paper and then put it in an oven at 100–105°C for 4 h. After it was transferred to a desiccator to cool for 30 min, it was weighed with an analytical balance. The drying step was repeated until the difference of the weights between the two drying steps was < .0003 g. Finally, we obtained weight of the dried residue (W). After adding 15% hydrogen peroxide solution, the residue continued to be heated in a water bath to remove organic matter. Then, the total amount of soluble salt (S) was obtained after drying. Salt content of the salt-alkali land was calculated as follows: soluble salt (%) = S (g)/W (g) ^*^100.

For determination of pH value of salt-alkali land, 10 g of the dry soil sample after being filtered by a 1-mm mesh was weighed and transferred into a 25-ml beaker. We added 10-ml.01 mol/L CaCl_2_ solution into the sample and mixed it well (Water: Soil = 1:1). And then the sample was placed for 10 min. pH value was measured with a pH meter (PHS-25, Shanghai Leici Instrument, Inc., Shanghai, China).

### Treatment Conditions and Experimental Design

**Figure 1:**
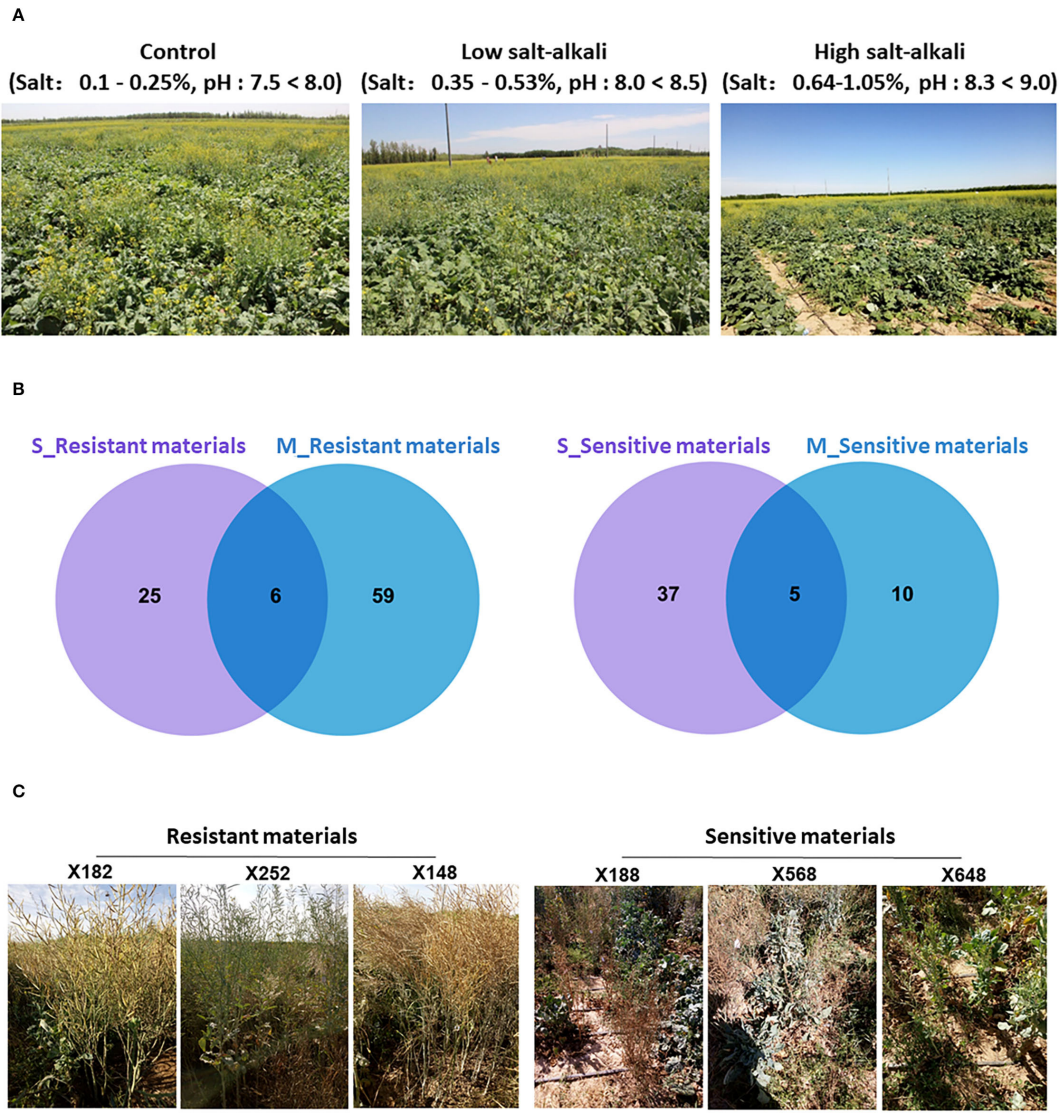
The plant growth stage of 505 *B. napus* accessions and screening results of extreme materials under salt-alkali stress conditions at seedling and mature stages. **(A)** Growth state of 505 *B. napus* accessions under control, low salt-alkali, and high salt-alkali conditions. **(B)** Venn diagrams showing resistant and sensitive materials under the salt-alkali condition at seedling and mature stages. **(C)** The response of extreme materials to salt-alkali stress at the mature stage in Inner Mongolia Autonomous Region of China.

Wuyuan County (105°12′-109°53′ E, 40°13′-42°28′ N) of Bayan Nur City is located in the western part of the Inner Mongolia Autonomous Region, which belongs to the hinterland of the Hetao Plain. The ingredients of saline-alkali land are dominated by sulfate, chloride, chloride-sulfate or sulfate-chloride in the Hetao Plain. We designed 3 treatment groups, including control (Salt:0.1–0.25%, pH: 7.5–8.), low salt-alkali (Salt:0.35–0.53%, pH: 8.−8.5), and high salt-alkali (Salt:0.64–1.05%, pH: 8.3–9.) conditions (Supplementary Figure 1). Each germplasm of 505 *B. napus* accessions was sown in 4 rows, and the space between each row was 20 cm. Each row contained 8 individual plants, and the space between each individual plant was 20 cm. At the same time, the second technical repetition was set up in the adjacent plots in different salt-alkali lands (Figure 1A). The sowing time is May 7, 2017. Before sowing, 225 kg/hm^2^ of complex fertilizer (N:P_2_O_5_:K_2_O, 25:10:16; total nutrients ≥ 51%; Shandong Stanley Fertilizer Co., Ltd.) was applied as base fertilizer to maintain normal growth and development of plants on May 7, 2017. In addition, 150 kg/hm^2^ of complex fertilizer as supplemental fertilizer was also applied on July 1, 2017. The irrigation was conducted on May 7, 2017, May 17, 2017, and July 1, 2017, respectively (Supplementary Table 30). We measured its salt content and pH value in control, low salt-alkali, and high salt-alkali stress conditions at seedling and mature stages (Supplementary Table 2). Then, some agronomic or growth indicators were determined (Supplementary Table 3) at the seedling (July 9, 2017) and harvest stages (September 9, 2017), respectively.

Shihezi City (84°58′-86°24′ E, 43°26′-45°20′ N) is located in the northern part of Xinjiang Uygur Autonomous Region, which belongs to the Southern margin of Junggar Basin. The ingredients of saline-alkali land are dominated by soda, chloride, sulfate-chloride, chloride-sulfate, or sulfate in Shihezi City. In order to further verify and screen extreme materials under salt-alkali condition, we designed 2 treatment groups, including control soil (Salt:0.05–0.15%, pH: 6.8–7.3), and high salt-alkali soil (Salt:0.80–1.15%, pH: 8.5–10). Each germplasm of 505 *B. napus* accessions was sown in 4 rows, and the space between each row was 20 cm. Eight individual plants were grown in each row for one replicate, and the space between each individual plant was 20 cm. At the same time, another technical repetition was conducted in the adjacent plots. The sowing date is July 10, 2018. Before sowing, 225 kg/hm^2^ of complex fertilizer (N:P_2_O_5_:K_2_O, 25:10:16; total nutrients ≥ 51%; Shandong Stanley Fertilizer Co., Ltd.) was applied as base fertilizer to maintain normal growth and development of plants on July 10, 2017. In addition, 150 kg/hm^2^ of complex fertilizer as supplemental fertilizer was also applied on August 4, 2017. The irrigation was conducted on July 10, 2017, August 4, 2017, and August 20, 2017, respectively (Supplementary Table 31). Then, we observed the response of the extreme materials at the seedling stage on September 19, 2018.

### Determination of Growth and Agronomic Indexes

Some traits, such as plant height (PH), ground dry weight (GDW), and yield (Y), were measured (Supplementary Table 4). For GDW measurement, materials were harvested at seedling and mature stages, respectively, and dried at 85°C for at least 72 h before the samples were weighed. We collected the traits data from 5 biological replicates for each accession (Lv et al., 2016; Yu et al., 2017). The tolerance coefficient (TC) was calculated as the ratio between the agronomic and growth indexes under salt-alkali stress and control condition (Supplementary Table 5).

### Screening Criteria of Extreme Materials

Combined with the response of these extreme materials to salt-alkali land in Xinjiang Uygur and Inner Mongolia Autonomous Region, the trait values of each variety were ranked, and then the top 10% most tolerant materials and the bottom 10% most sensitive materials were selected as extreme materials, respectively. Finally, a number of reliable extreme materials were screened out through Venn analysis between the two developmental stages.

### Statistical Analysis and Correlation Analysis

The statistically significant differences between different data sets were evaluated by the Student's *T*-test (*P* < 0.05). We finally calculated the average of all traits with 5 replicates per accession. We used the scale package of R language (https://www.r-project.org/) to analyze data standardization and R language stat.desc packages to analyze phenotypic variation. The correlation coefficient was calculated using the corr.test of psych package (https://www.r-project.org/), and figures were plotted using the corrplot package of R language (https://www.r-project.org/).

### Analysis of Genetic Effect, Treatment Effect, and Heritability

In order to determine the variance components, we used the mixed-effects variance analysis of the avo package by R language (https://www.r-project.org/), including genetic effects (G_effect), treatment effects (E_effect), and interaction effects between genetic and treatment effects (G&E effect). Heritability is a commonly used reference index in crop genetic breeding. The broad sense heritability (*H*_2_*b*) of all traits is calculated as follows: *H*_2_*b* = $\sigma_{G}^{2}$*/(*$\sigma_{G}^{2}$ + $\sigma_{GE}^{2}$*/n* + $\sigma_{e}^{2}$*/nr)* where $\sigma_{G}^{2}$ was the genotype variance, $\sigma_{e}^{2}$was the error variance, and $\sigma_{\text{GE}}^{2}$ was interaction between genetic and environmental effects. The estimates of *H2b* were analyzed by the AVOVA for ANOVA using the lmer function of the lme4 package by R environment (https://www.r-project.org/) (Chen et al., 2014; Li et al., 2020; Zhang et al., 2022).

### Genome-Wide Association Analysis (GWAS)

Our laboratory collected 505 *Brassica napus* germplasm resources to construct a re-sequencing association population (Tang et al., 2021). The high-quality clean reads data were obtained by the BWA (v0.75) software (https://github.com/lh3/bwa) (Li and Durbin, 2009). The reference genome is derived from “Brassica v4.1” (“Darmor-*bzh*”) genome (http://www.Genoscope.cns.fr/brassicanapus/data/). We also performed GWAS with 7,671,951 SNPs (MAF >0.05) using GEMMA linear mixed models (Zhou and Stephens, 2012). It is worth noting that we divided the gene components into 300 kb bins. If there are multiple suggestive SNPs in the same bin, it is considered to be located at the same site, and the suggestive SNP with the smallest *P*-value is regarded as the lead SNP.

### Identification of Candidate Genes and Polymorphisms

The *P*-value should be 1/marker number, 0.05/marker number or 0.01/marker number, which were named suggestive *P*-Value, significant *P*-value, and high-significant *P*-Value. High-density SNPs markers can improve the accuracy of candidate gene positioning, but there is often a certain linkage effect between adjacent SNP markers, so we often combined inter-linked SNP markers for one molecular marker. Therefore, we turned the final merged SNP markers, which were called effective SNP markers. The number of SNP markers used to calculate the *P*-value was far less than the total SNPs markers. Previous studies have shown that *B. napus* can be divided into two groups: spring and semi winter. In addition, semi winter was divided into Semi Winter 1 and Semi Winter 2 (Tang et al., 2021). The average number of SNP markers for different sub-species is about 7.38 million (Oberved_number) and 1.08 million (Effective_number), respectively. Effective SNPs account for about 14% of total SNPs. The average threshold is about 6 [~1. e-06, -log_10_ (*P*-value)] between different subspecies. This value was previously used by our laboratory in GWAS analysis of the same 505 *B. napus* accessions (Tang et al., 2021). Therefore, this value is usually considered as a recognized threshold in the 505 *B. napus* accessions. The suggestive and significant *P*-value threshold of the entire population was set to be 1. e-06. All SNPs exceeded the significance criterion were assessed for location of candidate genes. If coding regions were present, the potential impact on protein of each SNP was subsequently determined using the “Allele finder” facility Gene Ontology analysis. The candidate genes with differential gene expression ratios between the control treatment and stress treatment (R ≥ 2 or ≤ 0.5) (https://bigd.big.ac.cn/) (Zhang et al., 2019) were identified within 200 kb upstream or downstream of the lead SNP. We finally determined the protein function corresponding to the candidate gene in *Brassica napus* based on the homologous gene information on *Arabidopsis* (Chalhoub et al., 2014).

## Results

### Phenotypic Variation for Salt-Alkali Tolerance Traits to 505 *B. napus* Accessions at Seedling and Mature Stages

In order to evaluate the response of the 505 *B. napus* accessions to salt-alkali stress, we determined the related traits at seedling and mature stages under control (Salt:0.1–0.25%, pH: 7.5–8., CK), low salt-alkali (Salt:0.35–0.53%, pH: 8.−8.5, L), and high salt-alkali (Salt:0.64–1.05%, pH: 8.3–9., H) stress conditions, respectively (Figure 1A; Supplementary Figure 1). Plant height (S_PH_CK, S_PH_L, and S_PH_H) and aboveground dry weight (S_GDW_CK, S_GDW_L, and S_GDW_H) were measured at the seedling stage (Supplementary Table 4). In addition, the traits, such as plant height (M_PH_CK, M_PH_L, and M_PH_H), aboveground dry weight (M_GDW_CK, M_GDW _L, and M_GDW_H) and yield (M_Y_CK, M_Y_L, and M_Y_H) were obtained at the mature stage (Supplementary Table 4). We found that salt-alkali stress significantly inhibited the growth, development, and even yield of crops (Supplementary Figure 2).

Moreover, in order to better reflect the salt-alkali stress response, we also focused on the tolerance coefficients (TCs) of traits, which were calculated from the ratio of all traits between salt-alkali stress and control condition, denoted by the suffix type “trait_R1” under low salt-alkali stress conditions and “trait_R2” under high-salt-alkali stress conditions (Supplementary Table 5) (Guo et al., 2018). Descriptive statistics provided a good description of the phenotypic variation on 505 *B. napus* accessions under control condition and salt-alkali stress conditions. “Mean,” ‘Max,” “Min,” “SD,” “SE,” and “CV” were used to calculate the descriptive statistics of the average value of all traits (Supplementary Table 6). We found that the coefficient of variation (CV, %) of most traits reached more than 20%. Most traits of the 505 *B. napus* accessions show significant variations between control condition and salt-alkali stress conditions. Interestingly, large variations in plant height (M_PH), aboveground dry weight (M_GDW), and yield (M_Y) were observed among the accessions under salt-alkali stress conditions at the mature stage, whereas plant height (S_PH) and ground dry weight (S_GDW) showed small variations under the control condition at the seedling stage (Supplementary Table 6). These results suggest that the developmental stage has a big effect on the phenotypic variation under salt-alkali stress conditions.

After trait values of each strain were ranked, the top 10% most tolerant and bottom 10% most sensitive materials were selected as extreme materials, respectively. A number of reliable extreme materials have been screened out through Venn analysis between the two developmental stages (Figure 1B; Supplementary Figure 3). Combining the response of these extreme materials on salt-alkali land in Xinjiang Uygur and Inner Mongolia Autonomous Region of China, we finally identified that “X182,” “X252,” “X148,” “X200,” “X278,” and “X398” were as resistant varieties, and “X188,” “X568,” “X648,” “X552,” and “X756” were as sensitive varieties (Figure 1C; Supplementary Figure 4) under the two different salt-alkali stress environments. These extreme materials are valuable germplasm resources for the genetic improvement of salt-alkali tolerance in *B. napus*.

### Frequency Distribution and Correlation Analysis Among all Traits at Seedling and Mature Stages

**Figure 2:**
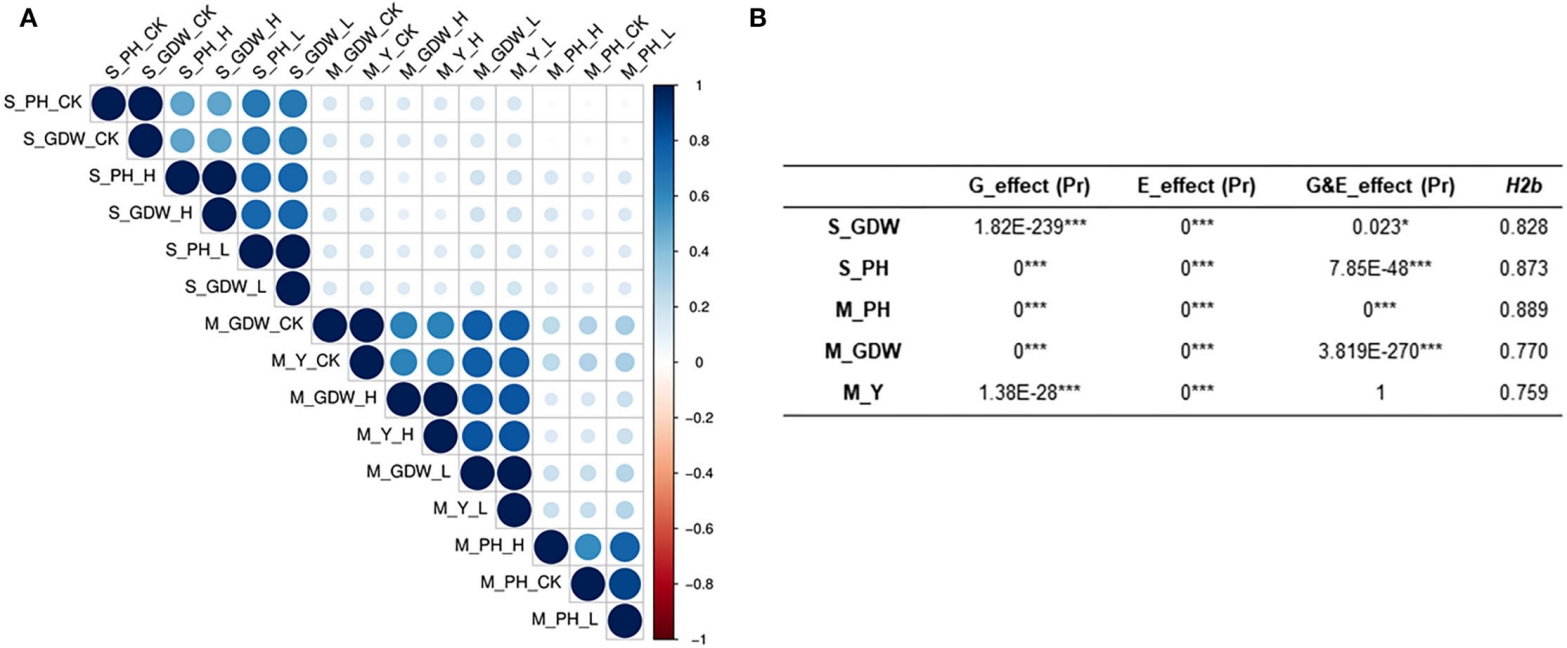
Evaluation of correlation coefficient, heritability, and genetic and treatment effects of all traits under salt-alkali stress conditions at seedling and mature stages. **(A)** Heatmap showing Pearson's correlations (*r*) among all traits of the 505 *B. napus* accessions assessed under control (CK, control condition, 0.1–0.25% salt content and pH 7.5–8.), low salt-alkali (L, low salt-alkali condition, 0.35–0.53% salt content, and pH 8–8.5), and high salt-alkali conditions (H, high salt-alkali condition, 0.64–1.05% salt content and pH 8.3–9.). S_GDW, ground dry weight and S_PH, plant height at the seedling stage. M_PH, plant height; M_GDW, ground dry weight; and M_Y, yield at the mature stage. **(B)** Broad-sense heritability, genetic and treatment effect analyses. *H2b*, broad-sense heritability; E_effect, treatment effect; G_effect, genetic effect; and G&E_effect, interaction of genetic and treatment effects. Statistical significance was evaluated by Student *T*-test. *p < 0.05 and ***p < 0.001.

We also performed normal distribution detection on the original absolute value (Supplementary Table 7) and TCs of all traits for GWAS (Supplementary Table 8), respectively. The absolute value of all traits conformed to normal or lognormal distributions and the TCs of all traits for GWAS also belonged to normal distributions. The lognormal distribution of S_PH and S_GDW may be attributed to the significant phenotypic variations among 505 *B. napus* accessions. Correlation analysis for all traits at seedling and mature stages was calculated and presented in Supplementary Table 9. The two traits at the seedling stage and the three traits at the mature stage showed significant correlations with “r” under control, low, and high salt-alkali stress conditions, which ranged from 0.021 to 0.982 between each other (Figure 2A; Supplementary Figures 5, 6; Supplementary Table 9). It is worth noting that the S_PH as strongly correlated with the S_GDW (*r* ≥ 0.5) and the M_GDW was strongly correlated with the M_Y (*r* ≥ 0.6), whereas the M_PH was weakly correlated with the M_GDW and M_Y (*r* ≥ 0.16) under control, low, and high salt-alkali stress conditions. Moreover, the strong positive correlations among control, low, and high salt-alkali stress conditions were observed for almost all the traits, and their correlations were from 0.497 to 0.869 while the correlation of all traits between seedling and mature stages was found to be relatively low. These results indicated that the genetic mechanism of salt-alkali stress response at the two different developmental stages was likely to be different.

### Heritability, Genetic, and Treatment Effect Analysis

Heritability is a key factor of decision-making in crop breeding, and it is essential for the effective design of breeding programs (Chen et al., 2014). Therefore, we also calculated the broad heritability (*H*_2_*b*) of all traits at seedling and mature stages. As shown in Supplementary Table 10, the variations in 505 *B. napus* accessions were largely determined by genotype and heritability estimates, which ranged from 0.759 to 0.889 (Figure 2B; Supplementary Table 10). Moreover, the genetic effect and treatment effect (G_effect and E_effect, *P* ≤ 0.05) could significantly represent the genotype effect and environment effect using the AVOVA by R environment (Figure 2B; Supplementary Table 10). Except for the S_GDW, the G&E_effects of other traits were found to be extremely significant. These results indicated that the response to salt-alkali stress is dynamic, which is largely determined by complex multiple genetic effects, and the mechanism of adaption to salt-alkali stress in *B. napus* is complicated.

### GWAS Revealed the QTLs Related to Salt-Alkali Tolerance in *B. napus* and Prediction of Candidate Genes

**Figure 3:**
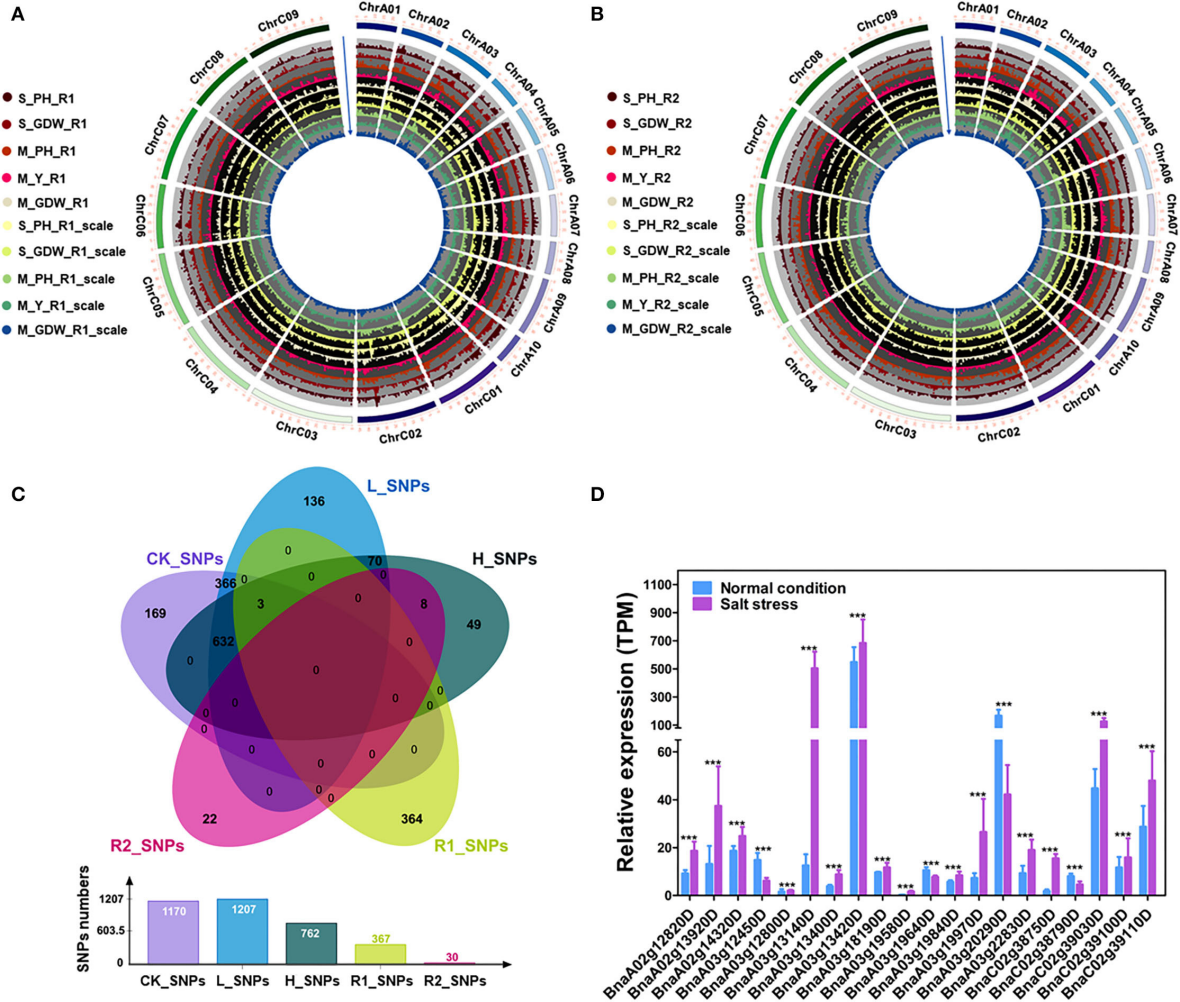
GWAS for tolerance coefficients (TCs) and identification of candidate genes. **(A)** Circle Manhattan plots with the co-localized SNPs for TCs of multiple traits under low salt-alkali condition (*P* ≤ 1e-2). S_PH_R1, the ratio of plant height between low salt-alkali and control conditions at the seedling stage; S_GDW_R1, the ratio of ground dry weight between low salt-alkali and control conditions at the seedling stage; M_PH_R1, the ratio of plant height between low salt-alkali and control conditions at the mature stage; M_Y_R1, the ratio of yield between low salt-alkali and control conditions at the mature stage; M_GDW_R1, the ratio of ground dry weight between low salt-alkali and slight control conditions at the mature stage; S_PH_R1_scale, normalized value of the plant height ratio between low salt-alkali and control conditions in the seedling stage; S_GDW_R1_scale, normalized value of the ground dry weight ratio between low salt-alkali and control conditions; M_PH_R1_scale, normalized value of the plant height ratio between low salt-alkali and control conditions at the mature stage; M_Y_R1_scale, normalized value of the yield ratio between low salt-alkali and control conditions at the mature stage; M_GDW_R1_scale, normalized value of the ground dry weight ratio between low salt-alkali and control conditions at mature the stage. **(B)** Circle Manhattan plots with the co-localized SNPs for TCs of multiple traits under the high-salt-alkali condition (*P* ≤ 1e-2). S_PH_R2, the ratio of plant height between high salt-alkali and control conditions at the seedling stage; S_GDW_R2, the ratio of ground dry weight between high-salt-alkali and control conditions at the seedling stage; M_PH_R2, the ratio of plant height between high-salt-alkali and control conditions at the mature stage; M_Y_R2, the ratio of yield between high-salt-alkali and control conditions at the mature stage; M_GDW_R2, the ratio of ground dry weight between high-salt-alkali and control conditions at the mature stage; S_PH_R2_scale, the ratio of normalized value of plant height between high-salt-alkali and control conditions at the seedling stage; S_GDW_R2_scale, the ratio of normalized value of ground dry weight between high-salt-alkali and control conditions at the seedling stage; M_PH_R2_scale, the ratio of normalized value of plant height between high-salt-alkali and control conditions at the mature stage; M_Y_R2_scale, the ratio of normalized value of yield between high-salt-alkali and control conditions at the mature stage; M_GDW_R2_scale, the ratio of normalized value of ground dry weight between high-salt-alkali and control conditions at the mature stage. **(C)** Co-localized SNPs under different environmental conditions revealed by Venn analysis. CK_SNPs, candidate SNPs of all traits under the control condition; L_SNPs, candidate SNPs of all traits under the low-salt-alkali condition; H_SNPs, candidate SNPs of all traits under high-salt-alkali conditions; R1_SNPs, candidate SNPs of all TCs under the low-salt-alkali condition; R2_SNPs, candidate SNPs of all TCs under the high-salt-alkali condition. The bar graph represents the number of SNPs under each environmental condition. **(D)** Gene expression of candidate differential expressed genes (DEGs) under salt stress condition. Statistical significance was determined by Student's *T*-test. ***p < 0.001.

The 15 absolute value and 10 TCs (Supplementary Tables 4, 5) were performed using the GEMMA linear mixed models for GWAS of all traits (Figures 3A,B; Supplementary Figures 7A–C) (Zhou and Stephens, 2012). About 3,536 (~0.046% of in total) strongly associated SNPs (–log_10_ (*P*-value) ≥ 6.) were chosen to further map the QTLs with the significant effect SNPs (Supplementary Table 11). A total of 1,170 SNPs (CK_SNPs), 1,207 SNPs (L_SNPs), and 762 SNPs (H_SNPs) were identified for absolute value of all traits under control, low salt-alkali, and high salt-alkali conditions, respectively. Moreover, 367 SNPs (R1_SNPs) and 30 SNPs (R2_SNPs) were identified for TCs under low salt-alkali and high salt-alkali conditions, respectively (Figure 3C; Supplementary Table 12). Based on these results, we obtained different numbers of specific and co-localized SNPs for different traits under different salt-alkali conditions. Among these SNPs, 740 specialized SNPs were detected, including 169 CK_SNPs, 136 L_SNPs, 49 H_SNPs, 364 R1_SNPs, and 22 R2_SNPs, respectively. About 366 co-localized SNPs (CK_SNPs and L_SNPs) and 9 co-localized SNPs (H_SNPs and R2_SNPs) were located on Chromosome A03, respectively. We also identified 70 (L_SNPs and H_SNPs), 632 SNPs (CK_SNPs, L_SNPs, and H_SNPs) and 3 co-localized SNPs (CK_SNPs, L_SNPs, H_SNPs, and R1_SNPs) located on Chromosome A10, respectively. Additionally, 20 co-localized SNPs on Chromosome A10 were simultaneously identified by M_GDW_L, M_PH_CK, M_PH_H, M_PH_L, and M_Y_L; 8 co-localized SNPs on Chromosome A03 were simultaneously identified by M_GDW_H, M_GDW_R2, M_Y_H, and M_Y_R2 (Figure 3C; Suppleentary Table 12). We finally obtained 9 main effect and co-localized QTLs related to salt-alkali stress response (Supplementary Table 13).

In order to identify the candidate genes associated with different traits under different salt-alkali stress conditions, we finally focused on the SNPs identified by GWAS for TCs of all traits, such as R1_SNPs (Figure 3C; Supplementary Tables 12, 14), R2_SNPs (Figure 3C; Supplementary Tables 12, 15), H_SNPs & R2_SNPs (Figure 3C; Supplementary Tables 12, 16), and CK_SNPs & L_SNPs & H_SNPs & R1_SNPs (Figure 3C; Supplementary Tables 12, 17). By analyzing the differential gene expression between control and stress conditions (*R* ≥ 2 or ≤ 0.5) (https://bigd.big.ac.cn/) (Zhang et al., 2019), 222 genes were identified within 200 kb upstream or downstream of the lead SNPs (Supplementary Table 18), such as 36 genes located on ChrA03 from 10,625,336 to 10,741,817 bp for M_Y_R1 and from 10,625,336 to 10,741,817 bp for M_GDW_R1 (Supplementary Figures 8A–C; Supplementary Table 14) and 15 candidate genes located on ChrA03 from 8,751,415 to 9,293,723 bp for M_Y_R2 and from 9,292,526 to 9,293,723 bp for M_GDW_R2 (Supplementary Figures 9A–C). GO analysis revealed that most genes were associated with the cellular process, the metabolic process, biological regulation, and response to stimulus (Supplementary Figure 10). Moreover, we also found that the most of these candidate genes were significantly differentially expressed under salt stress according to the RNA-seq data (PRJCA008229, https://ngdc.cncb.ac.cn/omix/) (Zhang et al., 2022). The relative expression of some candidate genes under normal and salt conditions was taken as an example (Figure 3D; Supplementary Table 19), which was consistent with the previous study (Zhang et al., 2019), showing the accuracy of candidate gene prediction. These results suggest that the accuracy of candidate gene prediction and the tolerance of *B. napus* to salt-alkali stress are regulated by a complex genetic mechanism.

### Prediction of Candidate Genes of Salt-Alkali Tolerance Within the Main Effect QTL *QSAT.A02.1* on ChrA02

**Figure 4:**
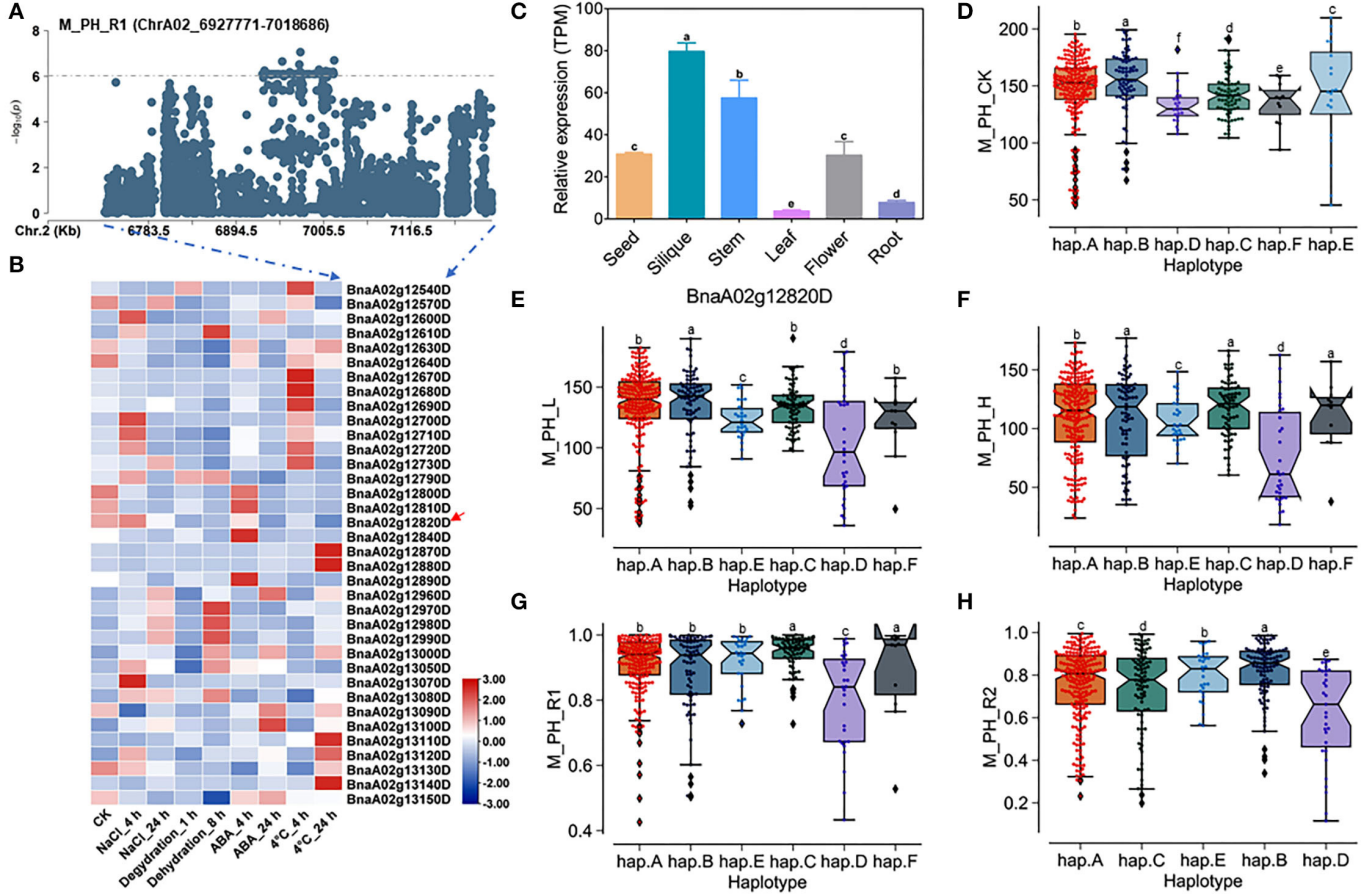
Analysis of candidate genes within the QTL *qSAT.A02.1* on ChrA02 associated with M_PH_R1. **(A)** A zoom-in view of the Manhattan plot on ChrA02 from 6,727,771 to 7,218,686 bp in the QTL *qSAT.A02.1* region. These points represented the log-transformed *P*-values from GWAS for the ratio of plant height between low-salt-alkali and control conditions at the mature stage (M_PH_R1). **(B)** Gene expression of candidate genes identified within 200 kb upstream or downstream of the significantly associated SNPs under 200-mM NaCl, 25-μM ABA, 4°C low temperature conditions for 4 and 24 h and under the dehydration condition for 1 and 8 h in *B. napus* cultivar Zhong Shuang 11 (“*ZS11*”). **(C)** Expression levels of BnA02g12820D in different tissues of *B. napus* cultivar “*ZS11*.” Values were means ± SD (*n* = 3 replicates), and different letters significantly indicate differences at *p* < 0.05 using two-way ANOVA. **(D–F)** Boxplots of haplotypes of BnA02g12820D for absolute value of the plant height (M_PH) at the mature stage under control conditions (M_PH_CK, **D**), the low-salt-alkali condition (M_PH_L, **E**), and under the high salt-alkali condition (M_PH_H, **F**). Values were means ± SD (*n* = 5 replicates), and different letters indicate significant difference at *p* < 0.05 using two-way ANOVA. **(G,H)** Boxplots of haplotypes of BnA02g12820D for relative value of the plant height (M_PH) at the mature stage under the low-salt-alkali condition (M_PH_R1, **G**) and under the high-salt-alkali condition (M_PH_R2, **H**). Values were means ± SD (*n* = 5 replicates), and different letters significantly indicate differences at *p* < 0.05 using two-way ANOVA.

A total of 36 genes within the QTL region on ChrA02 from 6,927,771 to 7,018,686 bp, which was named as to be *qSAT.A02.1* within 200 kb upstream and downstream of the candidate SNPs of M_PH_R1 (Figures 4A,B; Supplementary Tables 14, 18), and the expression of some genes were found to be significantly induced by many abiotic stresses (https://bigd.big.ac.cn/) (Zhang et al., 2019). Among these genes, we finally focused on the BnaA02g12820D, whose functions related to salt-alkali stress response have not been reported. BnaA02g12820D named as *BnABA4* is a homologous gene of *Arabidopsis ABA4*, which is involved in the photoprotection of PSII and plays a vital role in neoxanthin production in abscisic acid biosynthesis. Based on our RNA-seq data, we found that *BnABA4* was significantly induced by salt stress (Figure 3D). Moreover, the results from the expression pattern showed that *BnABA4* was highly expressed in siliques and stems (Figure 4C).

*BnABA4* consisting of five introns and six exons shows rich SNP variations, leading to synonymous and nonsense variation (Supplementary Table 20). The haplotypes of *BnABA4* were grouped into 6 haplotypes, including hap.A, hap.B, hap.C, hap.D, hap.E, and hap.F types (Supplementary Table 21). Both the absolute and relative values of plant height (M_PH) of hap.B type at the mature stage were significantly higher than other haplotypes under the control, low or high salt-alkali stress condition. We thus considered the accessions with hap.B types of *BnABA4* as extremely resistant haplotypes under salt-alkali stress conditions (Figures 4D–H). On the contrary, both the absolute and relative values of M_PH of hap.D type at the mature stage were significantly less than other haplotypes under control, low or high salt-alkali stress condition (Figures 4D–H). We thus considered the accessions with the hap.D types of *BnABA4* as extremely sensitive haplotypes under salt-alkali stress conditions. Therefore, *BnABA4* was considered as a candidate gene on the QTL *qSAT.A02.1* for salt-alkali stress response, showing a rich SNP variation in 505 *B. napus* accessions. The identified extreme haplotypes related to salt-alkali tolerance would be useful for breeding salt-alkali-tolerant *B. napus* in the future.

### Prediction of Candidate Genes of Salt-Alkali Tolerance Within the Main Effect QTL *QSAT.A02.2* on ChrA02

**Figure 5:**
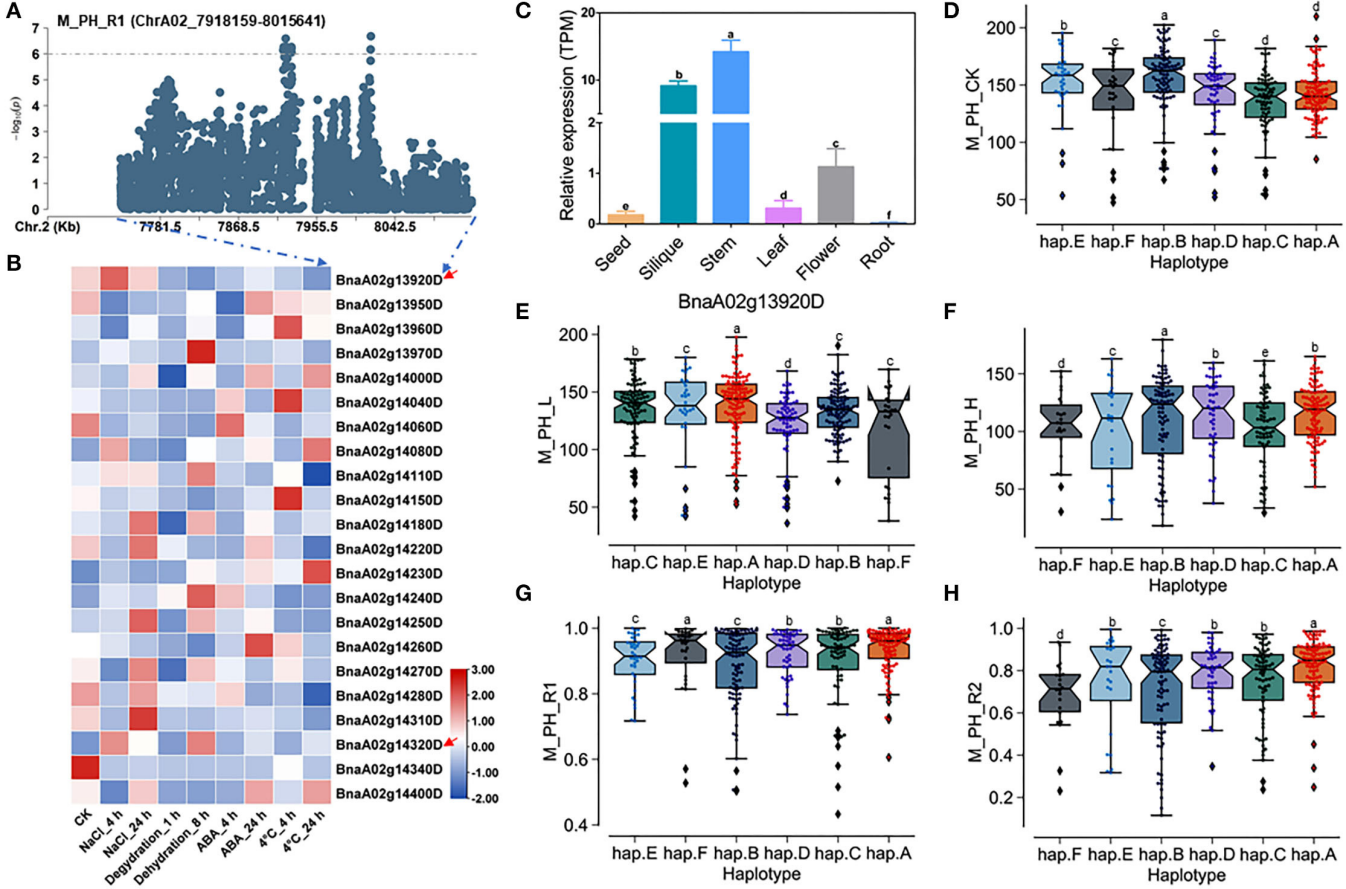
Analysis of candidate genes within the QTL *qSAT.A02.2* on ChrA02 associated with M_PH_R1. **(A)** A zoom-in view of the Manhattan plot on ChrA02 from 7,718,159 to 8,215,641 bp in the QTL *qSAT.A02.2* region. These points represented the log-transformed *P*-values for variants from GWAS for the ratio of plant height between low-salt-alkali and control conditions at the mature stage (M_PH_R1). **(B)** Gene expression of candidate genes identified within 200 kb upstream or downstream of the significantly associated SNPs under 200-mM NaCl, 25-μM ABA, 4°C low temperature conditions for 4 and 24 h and the dehydration condition for 1 and 8 h in *B. naps* cultivar “*ZS11*.” **(C)** Expression levels of BnA02g13920D in different tissues of *B. napus* cultivar “*ZS11*.” Values were means ± SD (*n* = 3 replicates), and different letters significantly indicate differences at *p* < 0.05 using two-way ANOVA. **(D–F)** Boxplots of haplotypes of BnA02g13920D for absolute value of the plant height (M_PH) at the mature stage under control conditions (M_PH_CK, **D**), the low-salt-alkali condition (M_PH_L, **E**), and under the high salt-alkali condition (M_PH_H, **F**). Values were means ± SD (*n* = 5 replicates), and different letters significantly indicate difference at *p* < 0.05 using two-way ANOVA. **(G,H)** Boxplots of haplotypes of BnA02g13920D for relative value of the plant height (M_PH) at the mature stage under the low-salt-alkali condition (M_PH_R1, **G**) and under the high-salt-alkali condition (M_PH_R2, **H**). Values were means ± SD (*n* = 5 replicates), and different letters significantly indicate differences at *p* < 0.05 using two-way ANOVA.

There were 22 genes within the QTL region on ChrA02 from 7,918,159 to 8,015,641 bp, which was named as to be *qSAT.A02.2* within 200 kb upstream and downstream of the candidate SNPs of M_PH_R1 (Figures 5A,B; Supplementary Tables 14, 18), and the expressions of some genes were found to be significantly induced by many stresses (https://bigd.big.ac.cn/) (Zhang et al., 2019). Among these genes, we finally focused on the BnaA02g13920D and BnaA02g14320D, whose functions related to salt-alkali stress have not been reported. BnaA02g13920D and BnaA02g14320D were named as *BnBBX14* and *BnVTI12*, which is the area homologous gene of *Arabidopsis BBX14* and *VTI12*, respectively. *BnBBX14* encodes a transcription factor of B-box zinc finger type with a CCT domain, which normally localizes to the transgolgi network. However, the *BnVTI12* belongs to the *VTI* gene family, which normally localizes to plasma membrane. Based on our RNA-seq data, we also found that *BnBBX14* and *BnVTI12* were significantly induced by salt stress (Figure 3D; Supplementary Table 19). Additionally, *BnBBX14* and *BnVTI12* were found to be expressed in various tissues. The expression level of *BnBBX14* was relatively high (Figure 5C) in siliques and stems. However, the expression level of *BnVTI12* in flowers was higher than other tissues (Supplementary Figure 11A).

*BnBBX14* consisting of one intron and two exons shows rich SNP sequence variations, leading to synonymous, missense, and disruptive inframe deletion variation (Supplementary Table 22). The haplotypes of *BnBBX14* were grouped into 6 haplotypes, including hap.A, hap.B, hap.C, hap.D, hap.E, and hap.F types (Supplementary Table 23). Except for hap.A types accessions of plant height (M_PH) under the control condition, both the absolute and relative values of M_PH of the hap.A type at the mature stage were significantly higher than other haplotypes under the control, low or high salt-alkali stress condition (Figures 5D–H). We thus considered the accessions with hap.A type of *BnBBX14* as extremely resistant materials under salt-alkali stress conditions. *BnVTI12* consisting of four introns and five exons exhibits rich SNP variations, leading to synonymous and splice region variants (Supplementary Table 24). The haplotypes of *BnVTI12* were grouped into 5 haplotypes, including hap.A, hap.B, hap.C, hap.D, and hap.E types (Supplementary Table 25). Both the absolute and relative values of plant height (M_PH) of the hap.E type at the mature stage were significantly higher than other haplotypes. We thus considered the accessions with hap.E type of *BnVTI12* as extremely resistant haplotypes under salt-alkali stress conditions. Except for M_PH trait absolute value of hap.A types accessions under control, low, and high salt-alkali stress condition, the relative values of hap.A type under low and high salt-alkali stress conditions were significantly higher than other haplotypes. We thus considered the accessions with hap.A type of *BnVTI12* as extremely resistant haplotypes under salt-alkali stress conditions. Therefore, *BnBBX14* and *BnVTI12* were considered as two candidate genes on *qSAT.A02.2* for salt-alkali stress response, showing a rich SNP variation among 505 *B. napus* accessions. The identified extreme haplotypes related to salt-alkali tolerance would be useful for breeding salt-alkali-tolerant *B. napus* in the future.

### Prediction of Candidate Genes of Salt-Alkali Tolerance Within the Main Effect of QTL *QSAT.A03.1* on ChrA03

**Figure 6:**
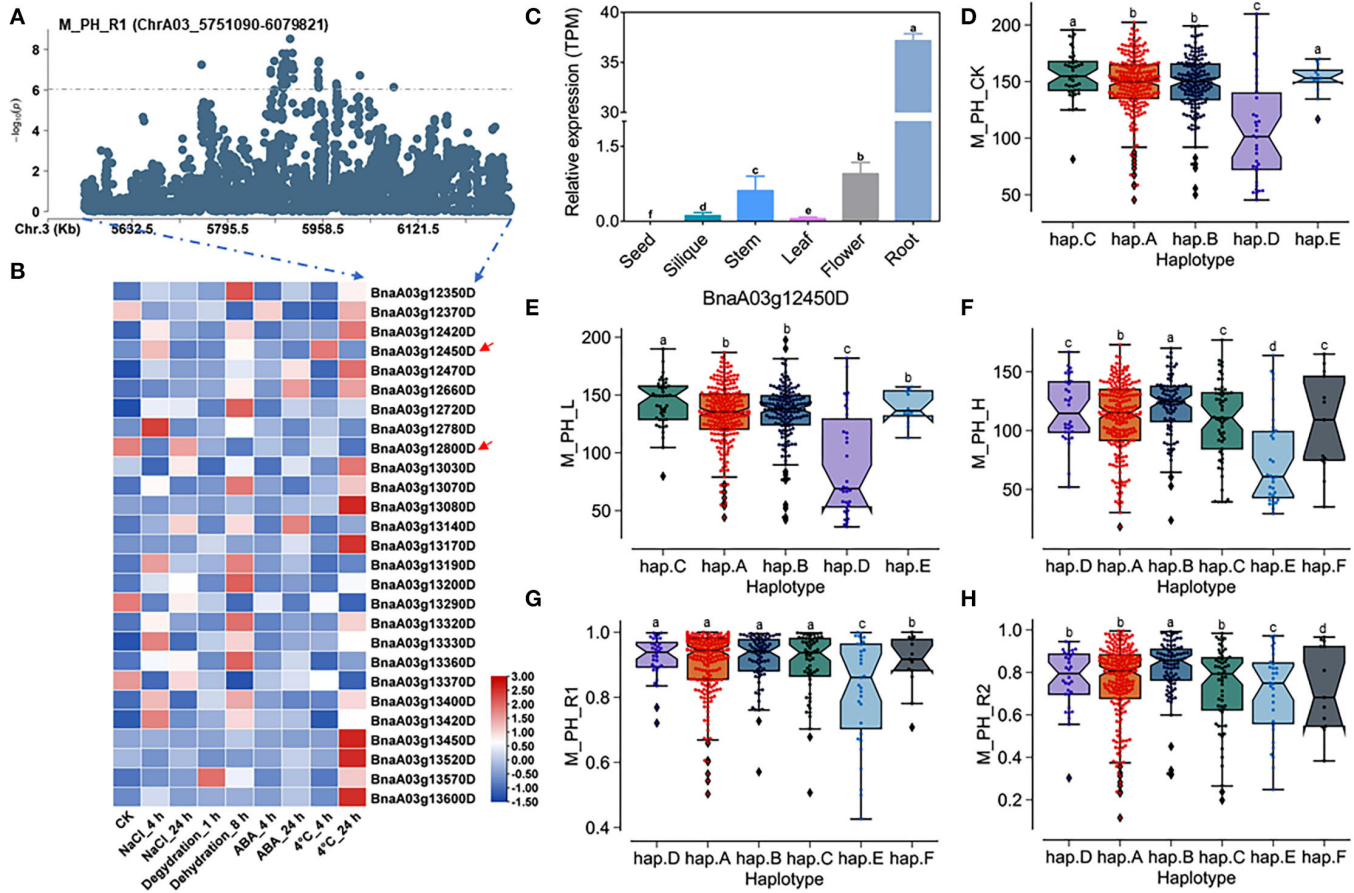
Analysis of candidate genes within the QTL *qSAT.A03* on ChrA03 associated with M_PH_R1. **(A)** A zoom-in view of the Manhattan plot on ChrA03 from 5,751,090 to 6,079,821 bp in the QTL *qSAT.A03* region. These points represented the log-transformed *P*-values for variants from GWAS of the ratio of plant height between low-salt-alkali and control conditions at the mature stage (M_PH_R1). **(B)** Gene expression of candidate genes identified within 200 kb upstream or downstream of the significantly associated SNPs under 200-mM NaCl, 25-μM ABA, 4°C low temperature conditions for 4 and 24-h dehydration conditions for 1 and 8 h in *B. napus* cultivar “*ZS11*.” **(C)** Expression levels of BnA03g12450D in different tissues of *B. napus* cultivar “*ZS11*.” Values were means ± SD (*n* = 3 replicates), and different letters significantly indicate differences at *p* < 0.05 using two-way ANOVA. **(D–F)** Boxplots of haplotypes of BnA03g12450D for absolute value of the plant height (M_PH) at the mature stage under control conditions (M_PH_CK, **D**), the low-salt-alkali condition (M_PH_L, **E**), and under the high-salt-alkali condition (M_PH_H, **F**). Values were means ± SD (*n* = 5 replicates), and different letters significantly indicate difference at *p* < 0.05 using two-way ANOVA. **(G,H)** Boxplots of haplotypes of BnA03g12450D for relative value of the plant height (M_PH) at the mature stage under the low-salt-alkali condition (M_PH_R1, **G**) and under the high-salt-alkali condition (M_PH_R2, **H**). Values were means ± SD (*n* = 5 replicates), and different letters significantly indicate differences at *p* < 0.05 using two-way ANOVA.

A total of 27 genes within the QTL region were identified on ChrA03 from 5,751,090 to 6,079,821 bp, which was named as to be *qSAT.A03.1* within 200 kb upstream and downstream of the candidate SNPs of M_PH_R1 (Figures 6A,B; Supplementary Table 14), and the expressions of some genes was found to be significantly induced by many stresses (https://bigd.big.ac.cn/) (Zhang et al., 2019). Among these genes, we finally focused on the BnaA03g12450D and BnaA03g12800D, whose functions related to salt-alkali stress response have not been reported. BnaA03g12450D and BnaA03g12800D were named as *BnPYL8* and *BnCRR1*, which are the homologous gene of *Arabidopsis PYL8/RCAR3* and *CRR1*, respectively. *BnPYL8* encoding an ABA receptor interacts with protein phosphatase 2Cs *ABI1* and *ABI2* in ABA signaling, and *BnCRR1* is involved in electron transfer between PSI and PSII and plays a vital role in the biogenesis or stabilization of the NAD(P)H dehydrogenase. Based on our RNA-seq data, we also found that *BnPYL8* and *BnCRR1* were significantly induced by salt stress (Figure 3D; Supplementary Table 19). In addition, *BnPYL8* and *BnCRR1* were found to be expressed in various tissues. The expression level of *BnPYL8* was relatively high in roots (Figure 6C), whereas the expression level of *BnCRR1* in the silique was higher than that in other tissues (Supplementary Figure 12A).

*BnPYL8* consisting of three introns and four exons exhibits rich SNP variations, leading to synonymous, missense, and splice region variation (Supplementary Table 26). The haplotypes of *BnPYL8* were grouped into 6 haplotypes, including hap.A, hap.B, hap.C, hap.D, hap.E, and hap.F types (Supplementary Table 27). Both the absolute and relative values of plant height (M_PH) at the mature stage in the hap.A, hap.B, and hap.C type accessions were significantly higher than other haplotypes, while those in the hap.E type accessions were significantly less than other haplotypes under the control, low or high salt-alkali stress condition. Both the absolute and relative values of plant height (M_PH) at the mature stage in the hap.A, hap.B, and hap.C type accessions were significantly higher than other haplotypes, while those in the hap.E type accessions were significantly less than other haplotypes under the control, low or high salt-alkali stress condition (Figures 6D–H). We could consider the accessions with hap.A, hap.B, and hap.C types of *BnPYL8* as extremely resistant haplotypes and the accessions with hap.E type of *BnPYL8* as extremely sensitive haplotypes under salt-alkali stress conditions. In addition, *BnCRR1* consisting of nine introns and ten exons shows rich SNP variations, leading to synonymous, missense, and splice region variation (Supplementary Table 28). The haplotypes of *BnCRR1* were grouped into 6 haplotypes, including hap.A, hap.B, hap.C, hap.D, hap.E, and hap.F types (Supplementary Table 29). Except for M_PH trait absolute value of hap.F types accessions under high salt-alkali stress conditions, both the absolute and relative values in the hap.F type accessions were significantly less than other haplotypes under control, low, and high salt-alkali stress conditions (Supplementary Figures 12B–F). We thus considered the accessions with hap.F type of *BnCRR1* as extremely sensitive haplotypes under salt-alkali stress conditions. On the contrary, both the absolute and relative values of plant height in the hap.A, hap.D, and hap.E type accessions were significantly higher than other haplotypes under control, low, and high salt-alkali stress conditions. We thus considered the accessions with hap.A, hap.D, and hap.E types of *BnCRR1* as extremely resistant haplotypes under salt-alkali stress conditions. According to the above results, *BnPYL8* and *BnCRR1* were considered as two candidate genes on *qSAT.A03.1* for salt-alkali stress response, which showed a rich SNP variation among 505 *B. napus* accessions. The identified extreme haplotypes associated with salt-alkali tolerance would be helpful for breeding salt-alkali-tolerant *B. napus* in the future.

## Discussion

Salt-alkali stress has significant influences on seedling establishment and yield performance (Zhao et al., 2020; Wassan et al., 2021). In this study, we determined the response to salt-alkali stress at seedling and mature stages in 505 accessions of *B. napus*. We totally obtained 2 phenotypic indexes at the seedling stage, including plant height and aboveground dry weight, and 3 growth traits, including plant height, aboveground dry weight, and yield at the mature stage under control, low, and high salt-alkali stress conditions, respectively (Supplementary Tables 2–4) (Batool et al., 2015).

We finally have screened out 11 reliable extreme materials at different developmental stages under the two different salt-alkali environments (Figures 1B,C; Supplementary Figures 3, 4). These materials may be useful for the genetic improvement of tolerance to salt-alkali stress in *B. napus*. In addition, it was found that the correlation coefficients between the various traits among different stress conditions at the same developmental stage were high, while the correlation coefficients between the same trait at seedling and mature stages were relatively low (Figure 2A; Supplementary Table 9). We also found that the plant height, aboveground dry weight, and yield are inhibited by salt-alkali stresses through the analysis of genetic effects (G_effect) and treatment effects (E_effect), and their broad-sense heritabilities (*H2b*) could better represent the genotypic effect of all traits, which were >0.75 (Figure 2B; Supplementary Table 10). These results suggest that the regulatory mechanism of salt-alkali stress responses at the seedling stage is likely to be different from that at the mature stage.

Previous studies have performed QTL mapping to explore the genetic basis of salt or salt-alkali stress responses at the germination or seedling stages (Yong et al., 2015; He et al., 2017; Hou et al., 2017; Wan et al., 2017, 2018; Zhang et al., 2017; Wassan et al., 2021). Although many QTLs and candidate genes related to salt or alkali stress response were identified in *B. napus*, no QTL or candidate gene has been reported to be associated with salt-alkali stress in *B. napus* until now. In this study, we finally chose 9 main-effect QTLs within 1,819 significant SNPs related to salt-alkali stress by GWAS of 15 absolute values and 10 TCs. We used the Venn analysis to identify co-localized QTLs and SNPs between different treatment conditions, developmental periods, and traits associated with salt-alkali stress response (Supplementary Tables 12, 13). In addition, we finally focused on the co-localized SNPs associated with the TCs of growth and agronomic traits (Supplementary Tables 13–17). Compared with the previous results, the two SNPs Bn-A03-p140441361 (ChrA03, site: 48,480 bp) and Bn-A06-p1015217 (ChrA06, site: 1,004,315 bp) previously identified by the GWAS for salt tolerance index of the germination rate (ST-GR) were co-localized with the QTL *qSAT.A03* on Chromosome A03 and *qSAT.A06* on Chromosome A06 according to our results, respectively. Moreover, the SNP Bn-A09-P1603989 (ChrA09, site: 2,323,836 bp) previously identified by the GWAS for salt tolerance index of fresh weight was co-localized with the QTL *qSAT.A09* on Chromosome A09 according to our results (Wan et al., 2018). Combining with expression analysis under various stress conditions, 222 candidate genes were identified (Supplementary Table 18) within 200 kb upstream and downstream of the candidate SNPs. Among these candidate genes, BnaA01g31420D (*BnBZIP28*), BnaA03g13820D (*BnWRKY25*), BnaA03g18900D (*BnCZF1*), BnaA03g18910D (*BnCZF1*), BnaA03g19270D (*BnCYSA*), BnaC02g39600D (*BnNHX1*), and BnaC02g39630D (*BnNHX1*) previously identified for root length, BnaA03g14410D (*BnSTH*) previously identified for the germination rate (He et al., 2017), BnaC03g22990D (*BnaUMAMIT11*) previously identified for hypocotyl length at seedling (HLW) and germination stages (HLN), and BnaA02g14680D (*BnRPK1*), BnaA02g14490D (*BnHVA22*), BnaC03g64030D (*BnCPL1*), and BnaC03g62830D (*BnMYB98*) previously identified for seedling dry weight and fresh weight by QTL Mapping (Hou et al., 2017) in previous studies on salt tolerance were also mapped in this study (Supplementary Table 18). Moreover, some identified candidate genes, such as BnaA02g12820D (*BnABA4*), BnaA02g14040D (*ABI3*), BnaA03g18230D (*BnCPK20*), BnaA03g12450D (*BnABA3*), BnaA03g12470D (*BnPP2C*), BnaA03g13820D (*BnWRKY25*), and BnaC02g38750D (*BnRAB*), have been reported to be related to salt stress responses. Interestingly, some previously unreported salt-stress-related genes were also identified, such as BnaA03g13140D (*BnLTP3*), BnaA03g19840D (*BnCBK3*), BnaA03g19970D (*BnPIF4*), BnaC02g38790D (*BnSAD1*), and BnaC02g39030D (*BnBXL1*) (Supplementary Tables 18, 19). Therefore, the GWAS approach was considered as a reliable and effective method to identify candidate genes related to salt-alkali stress.

Different from previous GWAS results, a larger population and high-density molecular markers were used in this study, which can improve the abundance of genetic variation within the population and the accuracy of candidate gene positioning. Moreover, compared with previous pieces of research on the salt stress response of plants grown in greenhouse at the germination and seedling stages, our study can truly reflect the response of *B. napus* to salt-alkali stress to *B. napus* under low and high salt-alkali land conditions. It was an important reference for the response of other crops to salt-alkali stress or other abiotic stresses through large-scale field trials on salt-alkali land. However, some shortcomings also existed in this study, such as uneven salt content of saline-alkali soil, a lack of more replicates in different years at different saline-alkali lands, and functional verification of candidate genes. Further studies are called for dissecting the complex genetic mechanism of salt-alkali tolerance in *B. napus*.

## Conclusion

Our results revealed significant natural variation in some growth and agronomic traits under salt-alkali stress. Using the GWAS approach, we not only confirmed the variation in alleles of some known salt tolerance-related genes but also identified some unreported genes or QTLs related to salt-alkali tolerance. An effective method was applied to identify some tolerant and sensitive accessions as valuable breeding materials for genetic improvement of salt-alkali tolerance of *B. napus*. This study has laid the foundation for understanding the complex genetic mechanism of salt-alkali tolerance and large-scale production of *B. napus* on salt-alkali land.

## Data Availability Statement

The datasets presented in this study can be found in online repositories. The names of the repository/repositories and accession number(s) can be found at: National Genomics Data Center (http://bigd.big.ac.cn), PRJCA008229.

## Author Contributions

XY, LG, GZha, and TF designed the research. GZha, YP, JZ, ZT, ChJ, SF, SZ, CuJ, RW, XW, BL, SL, and GZho performed the experiments or analyzed the data. GZha, LG, and XY analyzed the data and wrote the manuscript. All authors contributed to the article and approved the submitted version.

## Funding

This study was supported by Hubei Hongshan Laboratory Research Funding (2021HSZD004) and Higher Education Discipline Innovation Project (B20051).

## Conflict of Interest

The authors declare that the research was conducted in the absence of any commercial or financial relationships that could be construed as a potential conflict of interest.

## Publisher's Note

All claims expressed in this article are solely those of the authors and do not necessarily represent those of their affiliated organizations, or those of the publisher, the editors and the reviewers. Any product that may be evaluated in this article, or claim that may be made by its manufacturer, is not guaranteed or endorsed by the publisher.
