# Supplementary material for: Genome-Wide Association Studies of Salt-Alkali Tolerance at Seedling and Mature Stages in Brassica napus
Source: Front Plant Sci. 2022 Apr 27;13:857149. doi: 10.3389/fpls.2022.857149 (PMC9094488; doi:10.3389/fpls.2022.857149)
Supplement: Supplementary file 1 [file Table_1.DOCX]

**Additional information**

**Supplementary Figures**


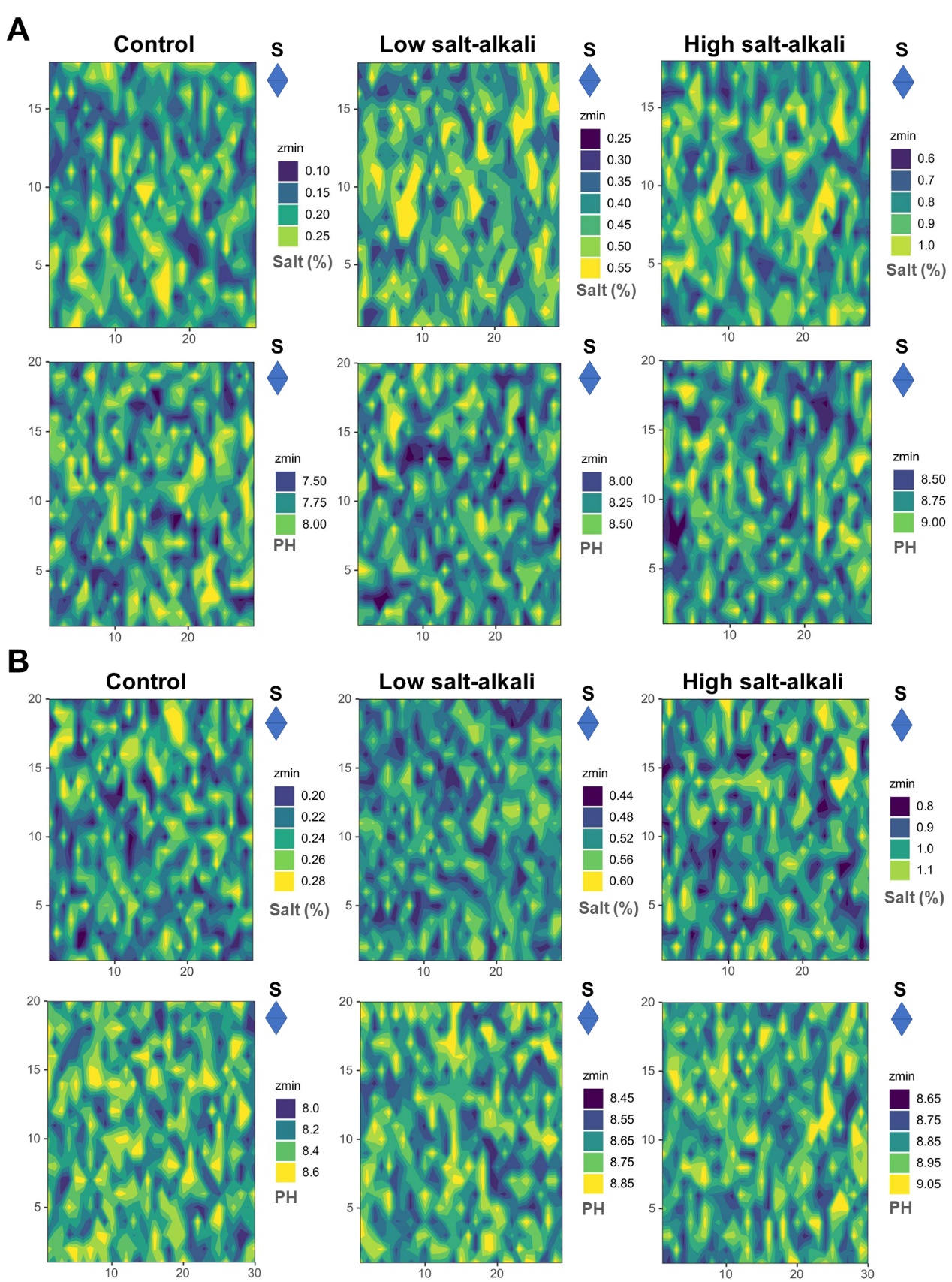


**Supplementary Figure 1.** Contour charts of salt-alkali distribution of control, low and high salt-alkali lands at seedling (**A**) and mature (**B**) stages.


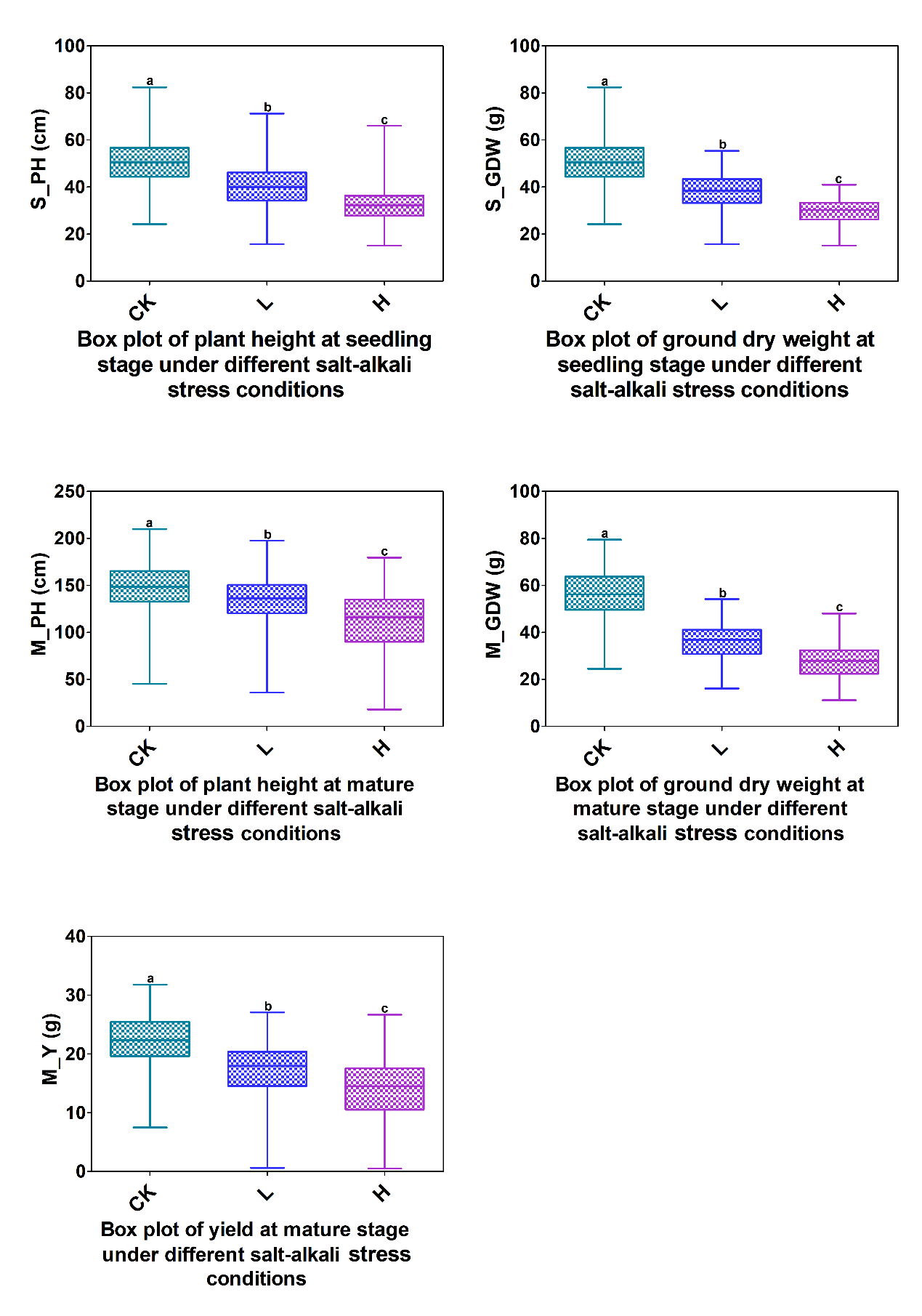


**Supplementary Figure 2.** Box plots of all traits under control (CK), low salt-alkali (L) and high salt-alkali (H) conditions at seedling and mature stages. Values were means ± SD (n = 5 replicates) and different letters significant indicate differences at P < 0.05 using two-way ANOVA.


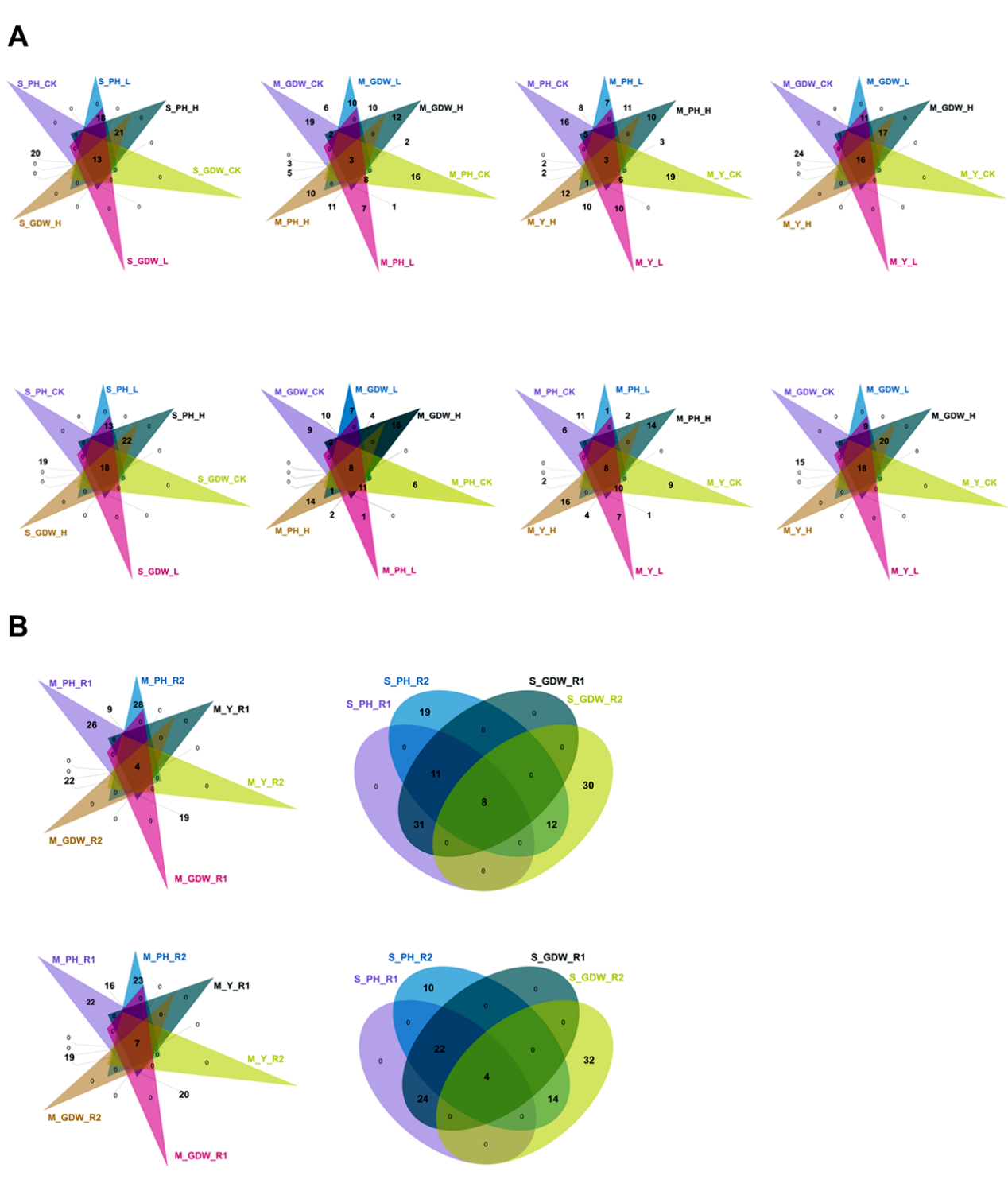


**Supplementary Figure 3.** Venn diagrams of extreme material screening process through the absolute value (**A**) and the ratio (**B**) between salt-alkali and control conditions at seedling and mature stages.


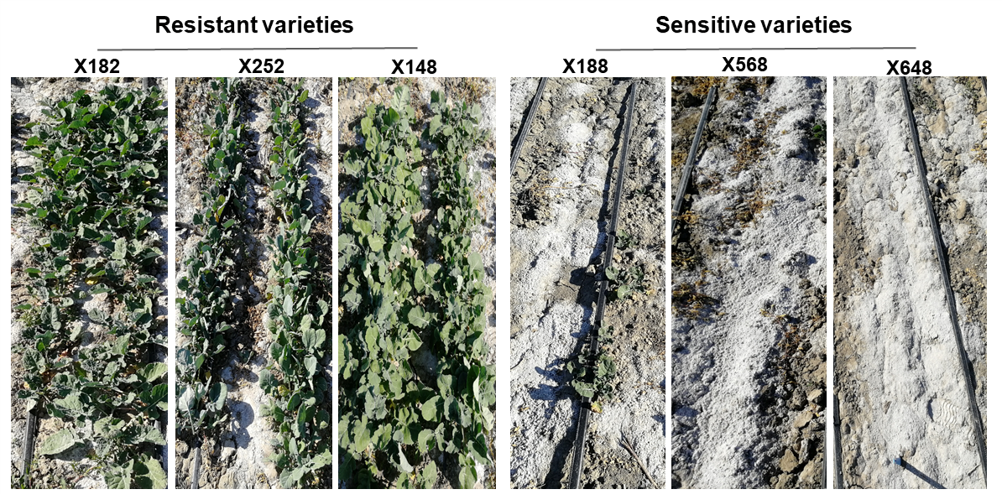


**Supplementary Figure 4.** Growth state of extreme materials at seedling stage under salt-alkali land in Xinjiang Uyghur Autonomous Region of China.

.
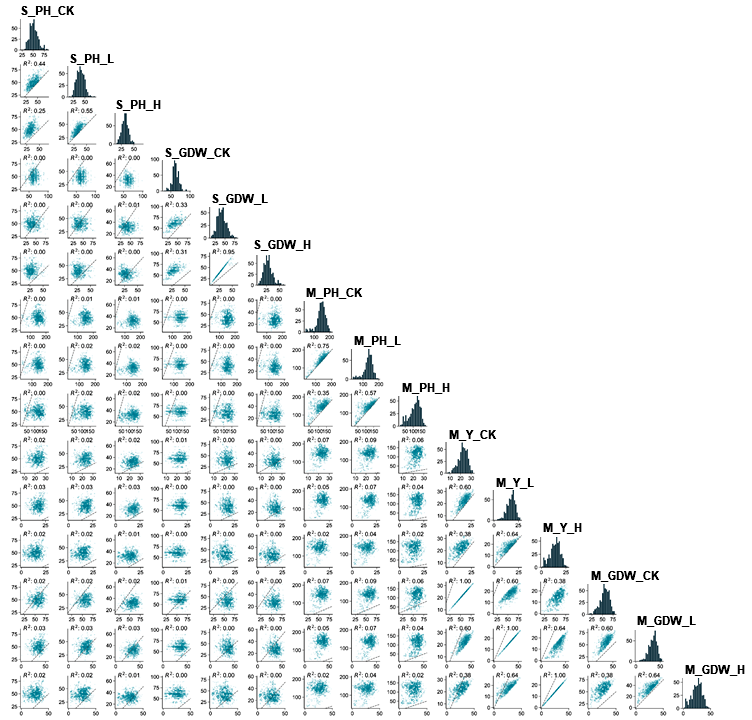


**Supplementary Figure 5.** Frequency distribution of all traits.


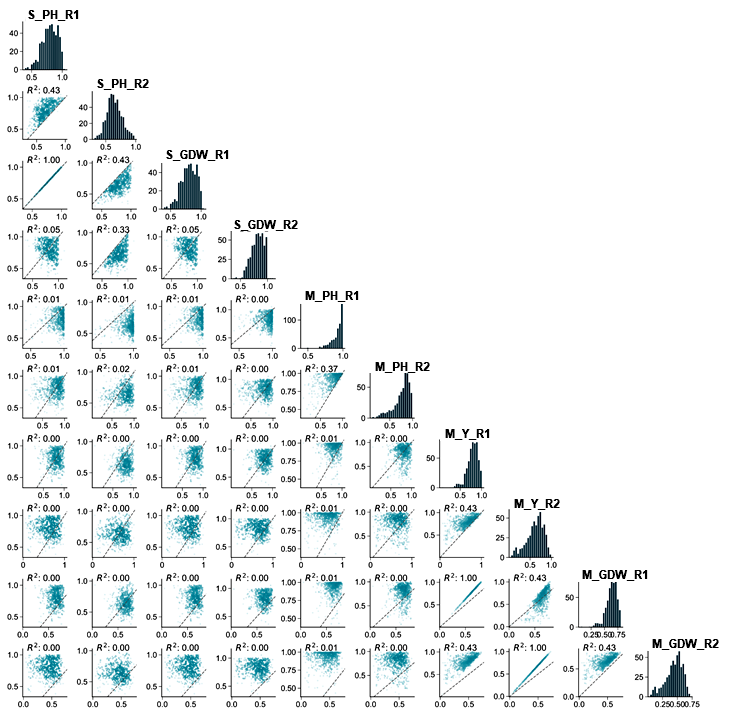


**Supplementary Figure 6.** Frequency distribution of tolerance coefficients (TCs) of all traits.


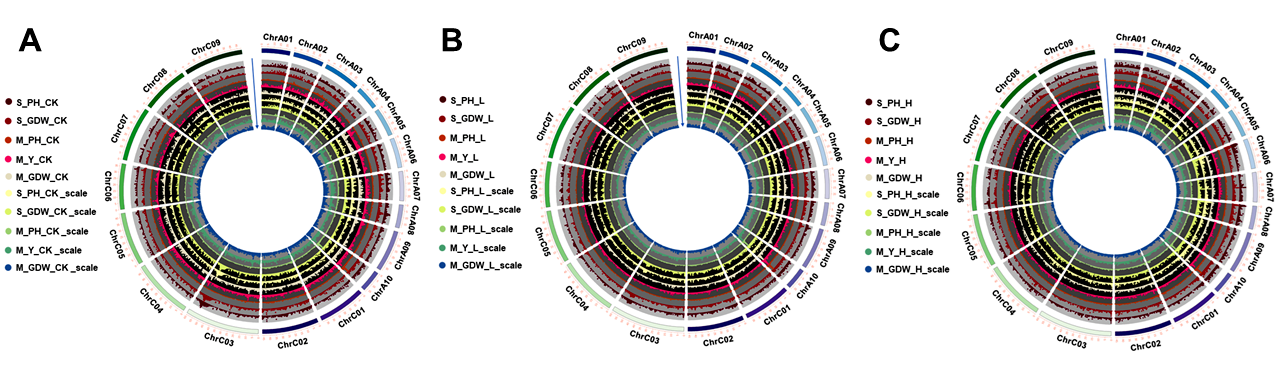


**Supplementary Figure 7**. Circle Manhattan plots by GWAS for absolute value of all traits.


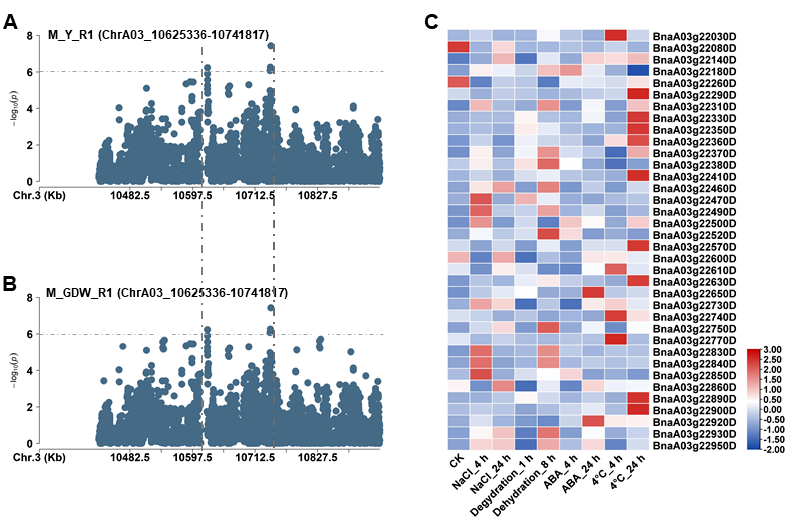


**Supplementary Figure 8.** Zoom-in view of the Manhattan plots on ChrA03 with co-localized loci identified by GWAS for M_Y_R1 (**A**) and M_GDW_R1 (**B**) and gene expression (**C**) of candidate genes identified within 200 kb upstream or downstream of the significantly associated SNPs under 200 mM NaCl, 25 µM ABA, 4 ℃ low temperature conditions for 4 h and 24 h and dehydration condition for 1 h and 8 h in *B. napus* cultivar ‘*ZS11*’.


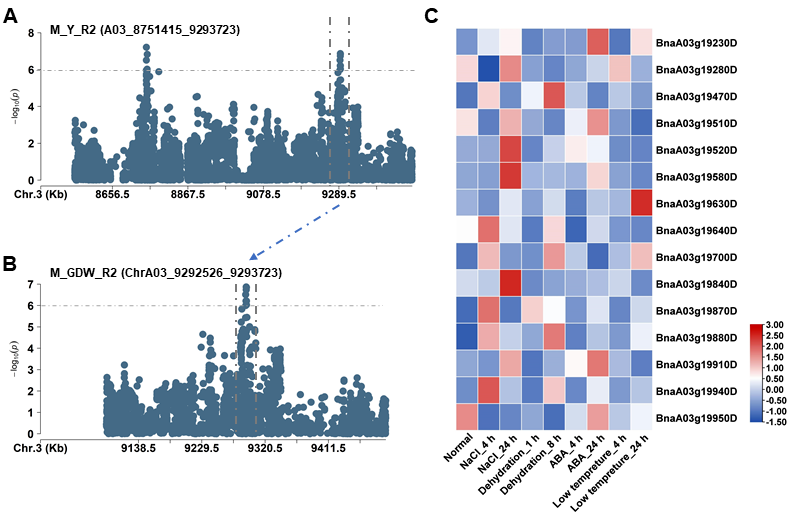


**Supplementary Figure 9.** Zoom-in view of the Manhattan plots on ChrA03 with co-localized loci identified by GWAS of M_Y_R2 (**A**) and M_GDW_R2 (**B**) and gene expression (**C**) of candidate genes identified within 200 kb upstream or downstream of the significantly associated SNPs under 200 mM NaCl, 25 µM ABA, 4 ℃ low temperature conditions for 4 h and 24 h and dehydration condition for 1 h and 8 h in *B. napus* cultivar ‘*ZS11*’.


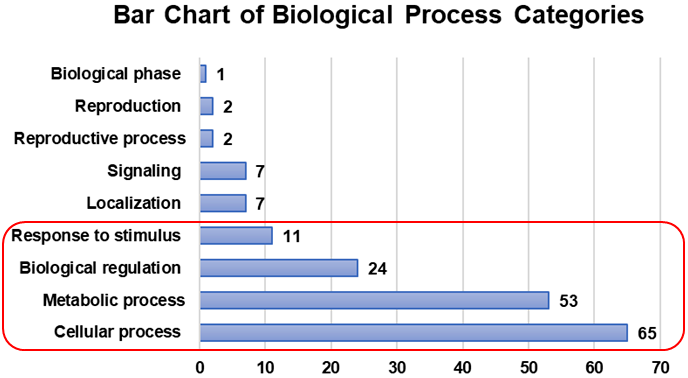


**Supplementary Figure 10.** GO enrichment analysis of candidate genes.


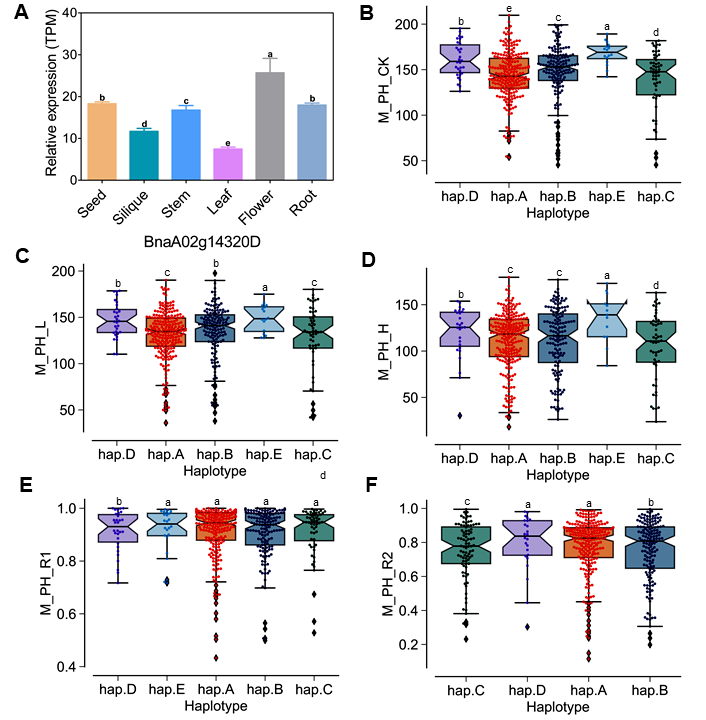


**Supplementary Figure 11.** Expression pattern (**A**) and haplotype analyses (**B-F**) of BnaA02g14320D. Values were means ± SD (n = 3, **A** or 5 replicates, **B-F**) and different letters significant indicate differences at P < 0.05 using two-way ANOVA.


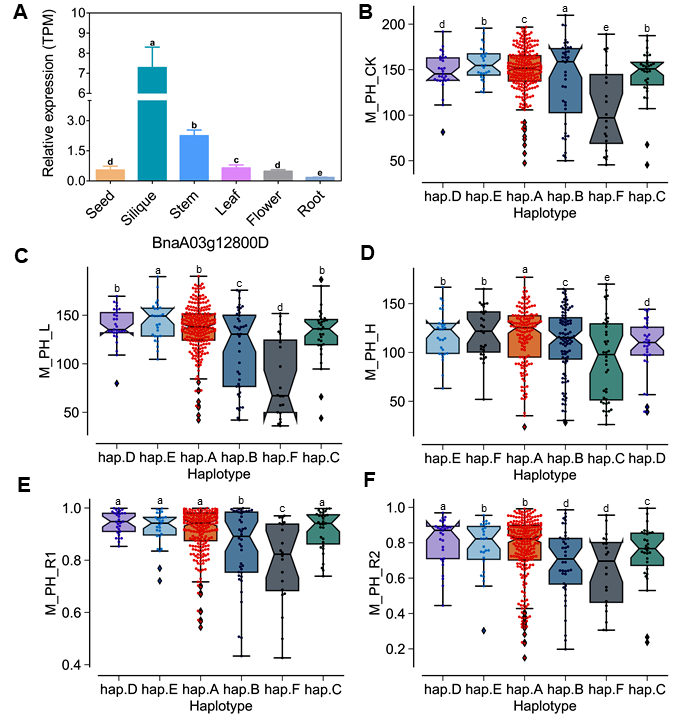


**Supplementary Figure 12.** Expression pattern (**A**) and haplotype analyses (**B-F**) of BnaA03g12800D. Values were means ± SD (n = 3, **A** or 5 replicates, **B-F**) and different letters significant indicate differences at P < 0.05 using two-way ANOVA.
